# Typical fast thermalization processes in closed many-body systems

**DOI:** 10.1038/ncomms10821

**Published:** 2016-03-01

**Authors:** Peter Reimann

**Affiliations:** 1 Fakultät für Physik, Universität Bielefeld, 33615 Bielefeld, Germany

## Abstract

The lack of knowledge about the detailed many-particle motion on the microscopic scale is a key issue in any theoretical description of a macroscopic experiment. For systems at or close to thermal equilibrium, statistical mechanics provides a very successful general framework to cope with this problem. However, far from equilibrium, only very few quantitative and comparably universal results are known. Here a quantum mechanical prediction of this type is derived and verified against various experimental and numerical data from the literature. It quantitatively describes the entire temporal relaxation towards thermal equilibrium for a large class (in a mathematically precisely defined sense) of closed many-body systems, whose initial state may be arbitrarily far from equilibrium.

## Abstract (web summary)

The relaxation of closed macroscopic systems towards thermal equilibrium is an ubiquitous experimental fact, but very difficult to characterize theoretically. Here, the author establishes a quantitative description of such relaxation under arbitrary typical conditions, capturing well experimental data.

## Graphical abstract

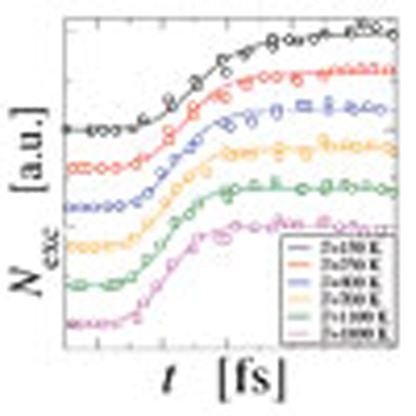

In a macroscopic object, which is spatially confined and unperturbed by the rest of the world, every single atom exhibits an essentially unpredictable, chaotic motion *ad infinitum*, yet the system as a whole seems to approach in a predictable and often relatively simple manner some steady equilibrium state. Paradigmatic examples are compound systems, parts of which are initially hotter than others, or a simple gas in a box, streaming through a little hole into an empty second box. While such equilibration and thermalization phenomena are omnipresent in daily life and extensively observed in experiments, they entail some very challenging fundamental questions: why are the macroscopic phenomena reproducible though the microscopic details are irreproducible in any real experiment? How can the irreversible tendency towards macroscopic equilibrium be reconciled with the basic laws of physics, implying a perpetual and essentially reversible motion on the microscopic level?

Such fundamental issues are widely considered as still not satisfactorily understood^1^,^2^,^3^,^4^,^5^,^6^. Within the realm of classical mechanics, they go back to Maxwell, Boltzmann and many others^7^. Their quantum mechanical treatment was initiated by von Neumann^8^ and is presently attracting renewed interest^9^,^10^,^11^,^12^, for example, in the context of imitating thermal equilibrium by single pure states due to such fascinating phenomena as concentration of measure^2^,^13^,^14^, canonical typicality^3^,^15^,^16^,^17^,^18^ or eigenstate thermalization^4^,^19^,^20^,^21^,^22^,^23^,^24^,^25^,^26^. Numerically, scrutinizing ultracold atom experiments^27^,^28^,^29^,^30^ and unravelling the relations between thermalization, integrability and many-body localization are among the current key issues^4^,^31^,^32^,^33^,^34^,^35^,^36^,^37^. Analytically, essential equilibration and thermalization properties of closed many-body systems or of subsystems thereof were deduced from first principles under increasingly weak assumptions about the initial disequilibrium, the system Hamiltonian and the observables^1^,^8^,^9^,^10^,^11^,^12^,^38^,^39^,^40^,^41^,^42^,^43^,^44^,^45^. In particular, groundbreaking results regarding pertinent relaxation timescales have been obtained in refs 45, 46, 47, 48, 49, 50, 51. Of foremost relevance for our present study is the work of the Bristol collaboration^49^, showing, among others, that all two-outcome measurements, where one of the projectors is of low rank, equilibrate as fast as they possibly can without violating the time–energy uncertainty relation. A second recent key result is due to Goldstein, Hara and Tasaki^50^,^51^, demonstrating that most systems closely approach an overwhelmingly large, so-called equilibrium Hilbert subspace on the extremely short Boltzmann timescale *t*_B_:=*h*/*k*_B_*T*. A more detailed account of pertinent previous works is provided as Supplementary Note 1.

Here we will further extend these findings in two essential respects: instead of upper bounds for some suitably defined characteristic timescale, as in refs 49, 50, 51, the entire temporal relaxation will be approximated in the form of an equality. As an even more decisive generalization of ref. 49, 50, 51, we will admit largely arbitrary observables. Finally, and actually for the first time within the realm of the above-mentioned analytical approaches^1^,^8^,^9^,^10^,^11^,^12^,^38^,^39^,^40^,^41^,^42^,^43^,^44^,^45^,^46^,^47^,^48^,^49^,^50^,^51^, we will compare our predictions with various experimental as well as numerical data from the literature. In fact, most of those data have not been quantitatively explained by any other analytical theory before. Adopting a ‘typicality approach' similar in spirit to random matrix theory^9^,^10^,^11^,^12^, our result covers the vast majority (in a suitably defined mathematical sense) of initial conditions, observables and system Hamiltonians. On the other hand, many commonly considered observables and initial conditions actually seem to be rather special in that they are close to or governed by a hidden conserved quantity and therefore thermalize ‘untypically slowly'.

## Results

### Set-up

Employing textbook quantum mechanics, we consider time-independent Hamiltonians *H* with eigenvalues *E*_*n*_ and eigenvectors |*n*〉 on a Hilbert space 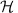 of large (but finite) dimensionality 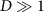. As usual, system states (pure or mixed) are described by density operators 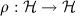 and observables by Hermitian operators 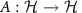 with matrix elements *ρ*_*mn*_:=〈*m*|*ρ*|*n*〉 and *A*_*mn*_:=〈*m*|*A*|*n*〉, respectively. Expectation values are given by 〈*A*〉_*ρ*_:=Tr{*ρA*} and the time evolution by 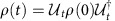 with propagator 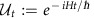, yielding


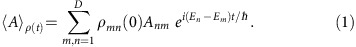


The main examples are closed many-body systems with a macroscopically well-defined energy, that is, all relevant eigenvalues *E*_1_, ..., *E*_*D*_ are contained in some microcanonical energy window [*E*−Δ*E*, *E*], where Δ*E* is small on the macroscopic but large on the microscopic scale. For systems with 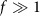 degrees of freedom, *D* is then exponentially large in *f* (refs 9, 41). Accordingly, the relevant Hilbert space 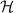 is spanned by the eigenvectors 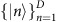 and is sometimes also named energy shell or active Hilbert space, see, for example, refs 8, 9, 10, 11, 12 and Supplementary Note 2 for more details.

### Analytical results

Our main players are the three Hermitian operators *H* (Hamiltonian), *A* (observable) and *ρ*(0) (initial state), each with its own eigenvalues (spectrum) and eigenvectors (basis of 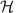). In the following, the three spectra will be considered as arbitrary but fixed, while the eigenbases will be randomly varied relatively to each other. More precisely, all unitary transformations 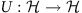 between the eigenbases of *H* and *A* are considered as equally likely (Haar distributed^8^,^9^,^10^,^11^), while the basis of *ρ*(0) relatively to that of *A* is arbitrary but fixed. (Equivalently, we could let ‘rotate' *H* relatively to *ρ*(0) while keeping *A* fixed relatively to *ρ*(0).) In particular, the initial expectation value 〈*A*〉_*ρ*(0)_ can be chosen arbitrary but then remains fixed (*U* independent). It is only for times *t*>0 that the randomness of the unitary *U* also randomizes (via *H*) the further temporal evolution of *ρ*(*t*) and thus of 〈*A*〉_*ρ*(*t*)_.

The basic idea behind this randomization of *U* is akin to random matrix theory^9^,^10^,^11^,^12^, namely, to derive an approximation for 〈*A*〉_*ρ*(*t*)_, which applies to the overwhelming majority of all those randomly sampled *U*'s, hence it typically should apply also to the particular (non-random) *U* of the actual system of interest. A more detailed justification of this ‘typicality approach' will be provided in the section ‘Typicality of thermalization'.

Since *A*_*mn*_ refers to the basis of *H*, these matrix elements depend on *U*, and likewise for *ρ*_*mn*_(0) (the explicit formulae are provided in the section ‘Basic matrices'). Indicating averages over *U* by the symbol [⋯]_*U*_ and exploiting that all basis transformations *U* are equally likely, it follows for symmetry reasons that [*ρ*_*nn*_(0)*A*_*nn*_]_*U*_ must be independent of *n*. Likewise, [*ρ*_*mn*_(0)*A*_*nm*_]_*U*_ must be independent of *m* and *n* for all *m*≠*n*. We thus can conclude that for any *n*


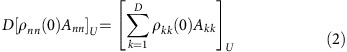


and that for any *m*≠*n*


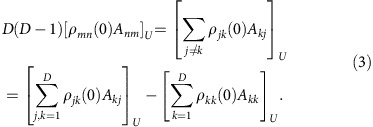


Defining the auxiliary density operator *ω* via the matrix elements *ω*_*mn*_:=*δ*_*mn*_*ρ*_*nn*_(0), equation (2) can be rewritten as [Tr{*ωA*}]_*U*_. Working in a reference frame where only *H* (and thus *ω*) changes with *U*, but not *A* and *ρ*(0), implies [Tr{*ωA*}]_*U*_=Tr{[*ω*]_*U*_*A*}. With *ρ*_av_:=[*ω*]_*U*_ it follows that


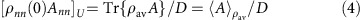


for arbitrary *n*. Likewise, equation (3) yields


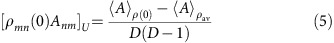


for arbitrary *m*≠*n*.

Upon separately averaging in equation (1), the summands with *m*=*n* and those with *m*≠*n* over *U*, and then exploiting equations (4) and (5) one readily finds that


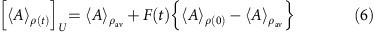



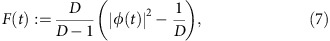


where *φ*(*t*) is the Fourier transform of the spectral density from ref. 46 (see also refs 51, 52, 53)


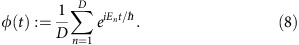


The following results can be derived in principle along similar lines (symmetry arguments being one key ingredient), but since the actual details are quite tedious, they are postponed to Methods. As a first result, one obtains


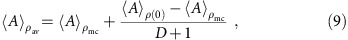


where *ρ*_mc_:=*I*/*D* is the microcanonical density operator and *I* the identity on 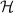. As a second result, one finds for the statistical fluctuations


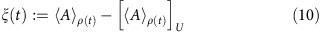


the estimate


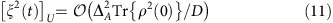


for arbitrary *t*, where Δ_*A*_ is the range of *A*, that is, the difference between the largest and smallest eigenvalues of *A*. Since averaging over *U* and integrating over *t* are commuting operations, equation (11) implies that


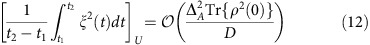


for arbitrary *t*_2_>*t*_1_.

Considering *t* in equation (11) as arbitrary but fixed, equation (10) and 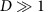 imply (obviously or by exploiting Chebyshev's inequality^1^,^9^,^40^,^43^,^45^) that 〈*A*〉_*ρ*(*t*)_ is practically indistinguishable from the average in equation (6) for the vast majority of all unitaries *U*. Indeed, the fraction (normalized Haar measure) of exceptional *U*'s is unimaginably small for typical macroscopic systems with, say, *f*≈10^23^ degrees of freedom, since *D* in equation (11) is exponentially large in *f* (see below equation (1)). Likewise, considering an arbitrary but fixed time interval [*t*_1_,*t*_2_] in equation (12), it follows for all but a tiny fraction of *U*'s that the time average over *ξ*^2^(*t*) on the left-hand side of equation (12) must be unimaginably small, and hence also the integrand *ξ*^2^(*t*) itself must be exceedingly small for the overwhelming majority of all *t*∈[*t*_1_,*t*_2_]. Accordingly, 〈*A*〉_*ρ*(*t*)_ must remain extremely close to equation (6) simultaneously for all those *t*∈[*t*_1_,*t*_2_].

Due to equation (9) and 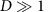, we furthermore can safely approximate 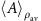 in equation (6) by 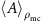. Altogether, we thus can conclude that in very good approximation


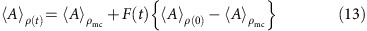


for the vast majority of unitaries *U* and times *t*. As detailed in Methods, the neglected corrections in equation (13) consist of a systematic (*U* independent) part, which is bounded in modulus by Δ_*A*_/(*D*^2^−1) for all *t*, and a random (*U* dependent) part (namely *ξ*(*t*)), whose typical order of magnitude is 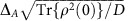 (for most *U* and *t*, cf. equations (11) and (12)), that is, *ξ*(*t*) is dominating by far (note that 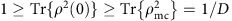). Moreover, the correlations of *ξ*(*t*) decay on timescales comparable to those governing *F*(*t*).

These are our main formal results. In the rest of the paper, we discuss their physical content.

### Basic properties of *F*(*t*)

Equation (8) implies that *φ*(0)=1, *φ*(−*t*)=φ*(*t*) and |*φ*(*t*)|≤1. With equation (7) and 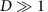, it follows that in very good approximation


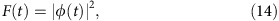


and thus


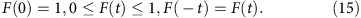


Indicating averages over all *t*≥0 by an overbar, one can infer from equations (8) and (14) that 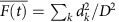, where *k* labels the eigenspaces of *H* with mutually different eigenvalues and *d*_*k*_ denotes their dimensions. Since ∑_*k*_ *d*_*k*_=*D*, we thus obtain 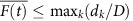. Excluding extremely large multiplicities (degeneracies) of energy eigenvalues, it follows that the time average 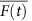 is negligibly small, and hence^1^,^9^,^40^,^43^,^45^ that *F*(*t*) itself must be negligibly small for the overwhelming majority of all sufficiently large *t*, symbolically indicated as


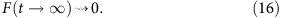


Note that there still exist arbitrarily large exceptional *t*'s owing to the quasi-periodicity of *φ*(*t*) implied by equation (8). We also emphasize that our main result (13) itself admits arbitrary degeneracies of *H*.

As an example, we focus on the microcanonical set-up introduced below equation (1) and on not too large times, so that equation (8) is well approximated by


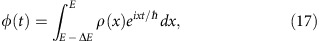


where *ρ*(*x*) represents the (smoothened and normalized) density of energy levels *E*_*n*_ in the vicinity of the reference energy *x*. If the level density is constant throughout the energy window [*E*−Δ*E*, *E*], we thus obtain with equation (14)


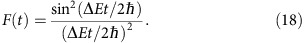


Next, we recall Boltzmann's entropy formula *S*(*x*)=*k*_B_ln (Ω(*x*)), where Ω(*x*) counts the number of *E*_*n*_'s below *x* and *k*_B_ is Boltzmann's constant. Hence, Ω′(*x*) must be proportional to the level density *ρ*(*x*) from above. Furthermore, *T*:=1/*S*′(*E*) is the usual microcanonical temperature of a system with energy *E* at thermal equilibrium. A straightforward expansion then yields the approximation 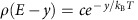 for *y*≥0, where *c* is fixed via 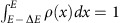. The omitted higher-order terms are safely negligible for all *y*≥0 and systems with 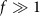 degrees of freedom, see also ref. 53. With equations (14) and (17), one thus finds


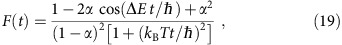


where 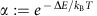. For 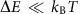, one recovers equation (18), and for 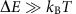, one obtains


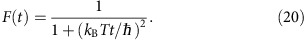


### Typicality of thermalization

Equations (13) and (16) imply thermalization in the sense that the expectation value 〈*A*〉_*ρ*(*t*)_ becomes (for most *U*) practically indistinguishable from the microcanonical average 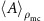 for the overwhelming majority of all sufficiently large *t*. Exceptional *t*'s are, for instance, due to quantum revivals, which, in turn, are apparently closely related to the quasi-periodicities of *F*(*t*).

Our assumption that energy eigenvalues must not be extremely highly degenerate (see above equation (16)) is similar to refs 46, 47, 49, 50, 51, but considerably weaker than the corresponding premises in most other related works^1^,^8^,^9^,^10^,^11^,^12^,^39^,^40^,^41^,^42^,^43^,^44^,^45^.

The usual time inversion invariance on the fundamental, microscopic level^7^ is maintained by equation (13) due to equation (15). Surprisingly, and in accordance with the second law of thermodynamics, the latter symmetry persists even if it is broken in the microscopic quantum dynamics, for example, by an external magnetic field.

By propagating *ρ*(0) backward in time (with respect to one particular *U*) and taking the result as new initial state, one may easily tailor^41^ examples of the very rare *U*'s and *t*'s, which notably deviate from the typical behaviour (13). Equivalently, one may back-propagate *A* instead of *ρ*(0) (Heisenberg picture).

Note that *S* and *T* were introduced below equation (18) not in the sense of associating some entropy and temperature to the non-equilibrium states *ρ*(*t*) but rather as a convenient level-counting tool. However, we now can identify them *a posteriori* with the pertinent entropy and temperature after thermalization.

The randomization via *U* (see the section ‘Analytical results') can be viewed in two ways: either one considers *ρ*(0), *A* and the spectrum of *H* as arbitrary but fixed, while the eigenbasis of *H* is sampled from a uniform distribution (Haar measure). Or one considers *H* and the spectra of *ρ*(0) and *A* as arbitrary but fixed and randomizes the eigenvectors of *A* and *ρ*(0). In doing so, a key point is that the relative orientation of the eigenbases of *ρ*(0) and *A* can be chosen arbitrarily but then is kept fixed. Indeed, it is well known^12^,^49^ that for ‘most' such orientations the expectation values 〈*A*〉_*ρ*(0)_ and 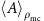 are practically indistinguishable, that is, an initial 〈*A*〉_*ρ*(0)_ far from equilibrium requires a careful fine-tuning of *ρ*(0) relatively to *A*.

In reality, there is usually nothing random in the actual physical systems one has in mind. Hence, results such as equation (13), which (approximately) apply to the overwhelming majority of unitaries *U*, should be physically interpreted according to the common lore of random matrix theory^9^,^10^,^12^, namely, as to apply practically for sure to a concrete system under consideration, unless there are particular reasons to the contrary.

Such reasons arise, for instance, when *A* is known to be a conserved quantity, implying a common eigenbasis of *A* and *H*, that is, the basis transformations *U* must indeed be very special. Furthermore, this non-typicality is structurally stable against sufficiently small perturbations of *A* and/or *H* so that the eigenvectors remain ‘almost aligned' (each eigenvector of *A* mainly overlaps with one or a few eigenvectors of *H*), and hence *A* remains ‘almost conserved' (almost commuting with *H*). Analogous non-typical *U*'s are expected when *ρ*(0) is known to be (almost) conserved (commuting with *H*).

Further well-known exceptions are integrable systems, for which thermalization in the above sense may be absent for certain *ρ*(0) and *A*^4^,^32^ (but not for others^22^), systems exhibiting many-body localization^34^,^36^ or trivial cases with non-interacting subsystems (Supplementary Note 2).

Our present focus is different: taking thermalization for granted is the temporal relaxation well approximated by equation (13)?

### Typical fast relaxation and prethermalization

Equation (20) is governed by the Boltzmann time *t*_B_:=*h*/*k*_B_*T*, amounting to *t*_B_≈10^−13^ s at room temperature. Equation (19) gives rise to comparably short timescales, unless the temperature is exceedingly low or the energy window Δ*E* is unusually small. Such relaxation times are much shorter than commonly observed in real systems^46^,^49^,^50^,^51^. Moreover, the temporal decay is typically non-exponential (see, for example, equations (18)–(20)), again in contrast to the usual findings.

This seems to imply that typical experiments correspond to non-typical unitaries *U*. Plausible explanations are as follows: to begin with, the above-predicted typical relaxation times are so short that they simply could not be observed in most experiments. Second (or as a consequence), the usual initial conditions and/or observables are indeed quite ‘special' with respect to the prominent role of almost conserved quantities (see previous section), in particular, ‘local descendants' of globally conserved quantities such as energy, charge, particle numbers and so on: examples are the amount of energy, charge and so on within some subdomain of the total system or, more generally, local densities, whose content within a given volume can only change via transport currents through the boundaries of that volume. As a consequence, the global relaxation process becomes ‘unusually slow' if the densities between macroscopically separated places need to equilibrate (small surface-to-volume ratio) or if there exists a natural ‘bottleneck' for their exchange (weakly interacting subsystems).

Put differently, our present theory is meant to describe the very rapid relaxation towards local equilibrium, but not any subsequent global equilibration. Only if there exists a clear-cut timescale separation between these two relaxation steps (or if there is no second step at all), can we hope to quantitatively capture the first step by our results. Conversely, the timescale separation usually admits some Markovian approximation for the second step, yielding an exponential decay, whose timescale still depends on many details of the system.

Natural further generalizations include the closely related concepts of hindered equilibrium, quasi-equilibrium (metastability) and, above all, prethermalization^29^,^54^,^55^, referring, for example, to a fast partial thermalization within a certain subset of modes, (quasi-)particles or other generalized degrees of freedom. (Like in ref. 54, we do not adopt here the additional requirement^55^ that the almost conserved quantities originate from a weak perturbation of an integrable system.)

In short, our working hypothesis is that the theory (13) describes the temporal relaxation of 〈*A*〉_*ρ*(*t*)_ for any given pair (*ρ*(0), *A*) unless one of them is exceptionally close to or in some other way slowed down by an (almost) conserved quantity.

### Comparison with experimental results

We focus on experiments in closed many-body systems in accordance with the above general requirements. In comparing them with our theory (13), we furthermore assume that the (pre-)thermalized system occupies a microcanonical energy window with some (effective) temperature *T* and 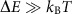, so that equation (20) applies. Finally, the asymptotic values 〈*A*〉_*ρ*(0)_ and 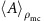 in equation (13) are either obvious or will be estimated from the measurements, hence no further knowledge about the often quite involved details of the experimental observables will be needed!

**Figure 1:**
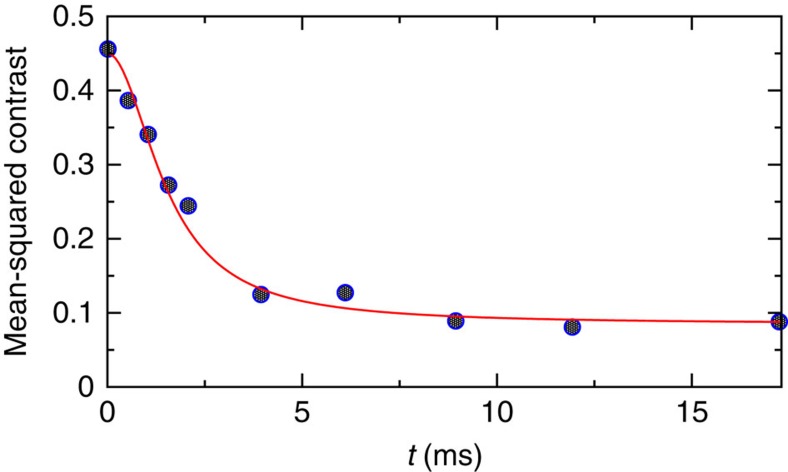
Prethermalization of ultracold atoms. The considered observable ‘mean-squared contrast' quantifies the spatial correlation of the matter–wave interference pattern after coherently splitting a Bose gas into two quasi-condensates (see ref. 29 for more details). Symbols: experimental data from Fig. 2a of ref. 29. Line: theoretical prediction (13) and (20) with *T*=5 nK. The pertinent effective temperature has also been roughly estimated in ref. 29 (see Fig. 2b therein) and is still compatible with our present fit *T*=5 nK. As discussed at the end of the section ‘Typical fast relaxation and prethermalization', the depicted prethermalization is followed by a much slower, global thermalization^29^, which is omitted in the present figure.

Figure 1 demonstrates the very good agreement of the theory with the rapid initial prethermalization of a coherently split Bose gas, observed by the Schmiedmayer group in ref. 29.

**Figure 2:**
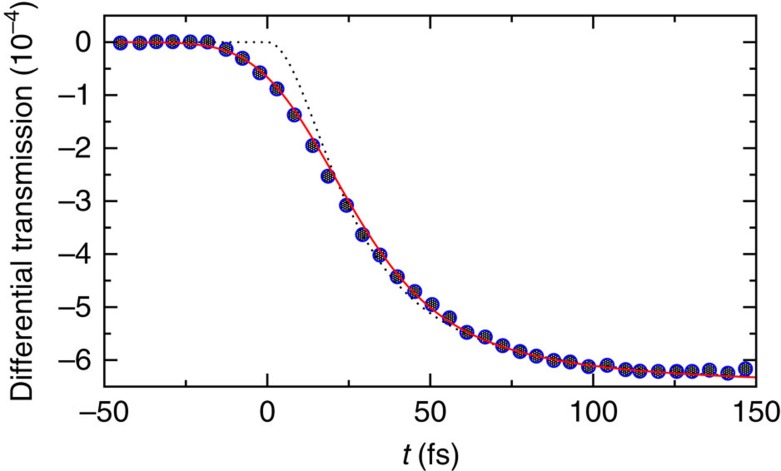
Ultrafast relaxation of hot electrons. A first laser pulse (at *t*=0) ‘heats up' the electron gas in a thin ferromagnetic film, whose rethermalization is then probed by means of a second laser pulse. As detailed in ref. 56, the considered observable ‘differential transmission' quantifies the magneto-optical polarization rotation of the probe laser light. Symbols: experimental data from Fig. 2a of ref. 56. Dotted: theoretical prediction (13) and (20) with *T*=310 K and *F*(*t*<0):=1. Solid: convolution of the dotted line with a Gaussian of 35 fs FWHM, accounting for the finite widths of the pump and probe laser pulses (see also main text). Similarly as in Fig. 1, on larger timescales than covered by the present figure, the prethermalized electrons also exhibit non-negligible interactions with the lattice phonons and magnons, resulting in a much slower global relaxation of the compound electron–lattice system^56^. Concerning the pertinent temperature *T*, a direct experimental estimate is not available for the set-up from ref. 56 (I contacted one of the authors), but it has been provided for a similar experiment by the same group in ref. 65, except that the fluence (energy per spot area of the pump laser pulse) was 70 times larger than in ref. 56. Taking all this into account, the estimate *T*=310 K adopted in the present figure seems very reasonable.

In Fig. 2, the theory is compared with the pump-probe experiment by the Bigot group from ref. 56. The finite widths of the pump and the probe laser pulses are roughly accounted for by convoluting equation (13) with a Gaussian of 35 fs FWHM (full width at half maximum). In ref. 56, the FWHM of the pump pulse is estimated as 20 fs and the combined FWHM for both pulses as 22 fs, implying a FWHM of 9 fs for the probe pulse. The latter value seem quite optimistic to us. A second ‘excuse' for our slightly larger FWHM value of 35 fs is that the tails of the experimental pulse shape may be considerably broader than those of a Gaussian with the same FWHM (see, for example, Fig. 2c in the Supplementary Material of ref. 57). Finally, the convolution of equation (13) with a Gaussian represents a rather poor ‘effective description' in the first place: our entire theoretical approach becomes strictly speaking invalid when the duration of the perturbation becomes comparable to the thermalization time.

**Figure 3:**
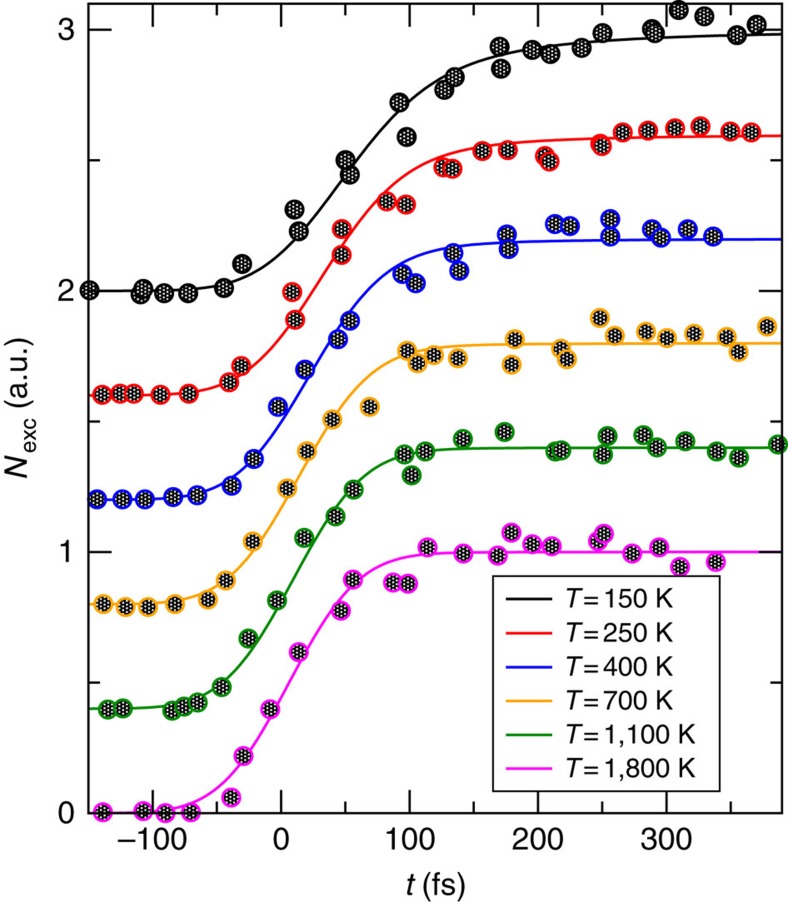
Temperature-dependent relaxation of hot electrons. Symbols: similar pump-probe experiments as in Fig. 2, but now conducted on bismuth and for six different fluences (energy per spot area of the pump laser pulses). As detailed in ref. 58, the considered observable *N*_exc_ quantifies (in a.u.) the number of excited electrons above the Fermi level. The depicted data are from Fig. 5b of ref. 58 for fluences (top–down) 0.12, 0.2, 0.36, 0.52, 0.68, and 0.84 mJ cm^−2^. Lines: theoretical prediction (13) and (20) with temperatures as indicated and convoluted with a Gaussian of 100 fs FWHM (see also main text). The conversion of a given fluence into a temperature change of the electron gas is not obvious. In particular, the estimates provided in ref. 58 seem not very reliable to us: first of all, Fig. 6 in ref. 58 indicates a temperature of ca. 250 K at four different time points ∼200 fs before the pump pulse, while the actual temperature of the unperturbed system is known to be 130 K. Second, the temperature error bars in Fig. 6b of ref. 58 are quite large. Third, a key premise of those estimates in ref. 58 is that the ‘renormalized' curves in Supplementary Fig.3b of ref 66 should coincide, while their actual agreement is only moderately better than for the ‘bare' curves in Supplementary Fig. 3a. For all these reasons, we used the temperature as a fit parameter in the present figure.

A similar comparison with the pump-probe experiments from ref. 58 is presented in Fig. 3. As before, we adopted a slightly larger FWHM of 100 fs than the estimate of 76 fs in ref. 58. Due to the above-mentioned fundamental limitations of our theory for such rather large FWHM values, the temperatures adopted in Fig. 3 should still be considered as quite crude estimates. Apart from that, Fig. 3 nicely confirms the predicted temperature dependence from equation (20).

We close with three remarks: first, refs 56, 58 also implicitly confirm our prediction that the essential temporal relaxation (encapsulated by *F*(*t*) in equation (13)) is generically the same for different observables. Second, similar pump-probe experiments abound in the literature, but usually the pulse widths are too large for our purposes. Third, the temporal relaxation in Figs 1, 2, 3 has also been investigated numerically, but closed analytical results have not been available before^29^,^58^.

### Comparison with numerical results

**Figure 4:**
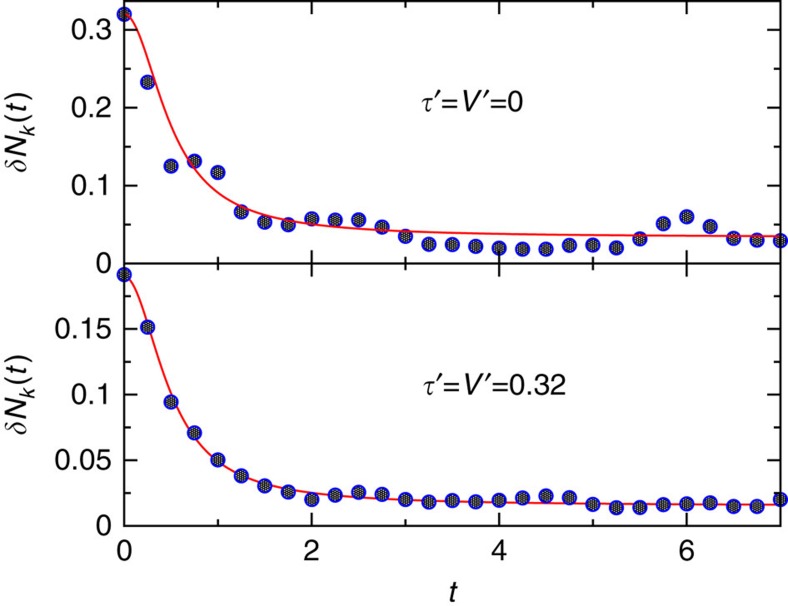
Relaxation of an integrable and a non-integrable fermionic model. The upper part of the plot refers to an integrable model, the lower to a nonintegrable one. Symbols: numerical results from ref. 32 for eight strongly correlated fermions on a one-dimensional lattice with 24 sites, described in terms of an extended Hubbard model with nearest- and next-nearest-neighbour hopping and interaction parameters *τ*, *τ*′, *V* and *V*′, respectively. Working in units with *ℏ*=*k*_B_=*τ*=*V*=1 and focusing on parameters *τ*′=*V*′, the model is integrable if *τ*′=*V*′=0 and non-integrable otherwise. A quantum quench generates an initial pure state out of equilibrium, whose energy corresponds to that of a canonical ensemble with temperature *T*=2. As detailed in ref. 32, the considered observable *δN*_*k*_(*t*) is a dimensionless descendant of the density–density structure factor. The depicted data are from Fig. 1g,j of ref. 32. Lines: theoretical predictions (13) and (20) with *T*=2.

Figure 4 illustrates the very good agreement of our theory with Rigol's numerical findings from ref. 32, both for an integrable and an non-integrable example. A similar agreement is found for all other parameters and also for an analogous hardcore boson model examined in refs 31, 32. On the other hand, a second observable considered in ref. 32, deriving from the momentum distribution function, exhibits in all cases a significantly slower and also qualitatively different temporal relaxation. According to the discussion in the section ‘Typical fast relaxation and prethermalization', it is quite plausible that the latter observable is indeed ‘non-typical' in view of the fact that it represents a conserved quantity for fermions with *V*=*τ*′=*V*′=0 (ref. 32).

**Figure 5:**
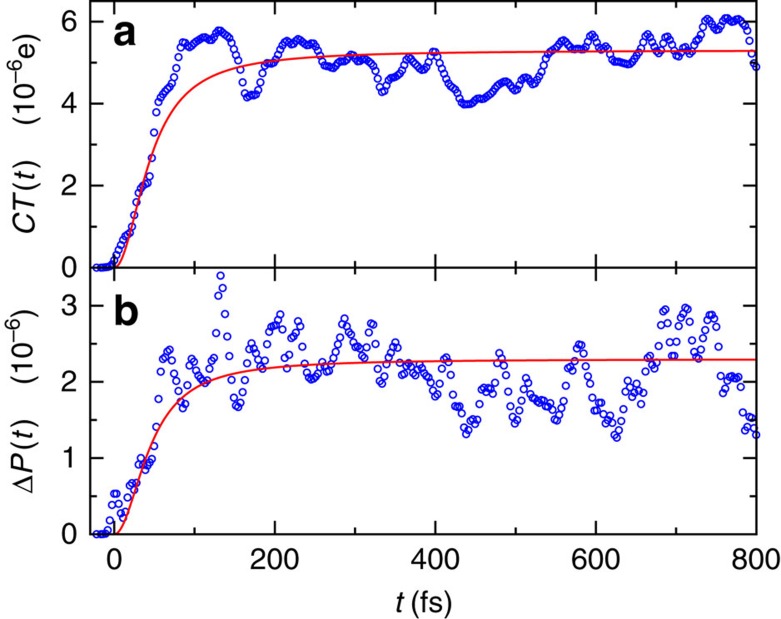
Prethermalization in a one-dimensional electron gas. Symbols: numerical results from ref. 59 for a one-dimensional model of the many-electron dynamics in an asymmetric double-well potential (emulating a metal–insulator–metal junction). Starting with 44 electrons in the ground state, a laser pulse-like electrical perturbation acts predominantly on the 16 electrons in the smaller, box-shaped well, and then their rethermalization is monitored via the charge transfer into the larger well (denoted in (a) as *CT*(*t*)), and via the change of the ground state population (denoted in (b) as Δ*P*(*t*)). Depicted are the numerical results from Fig. 8 of ref. 59. For further details regarding the simulations, we refer to refs 59, 67. Lines: theoretical predictions (13) and (20), exploiting the estimate *T*=170 K from the main text, and neglecting the finite temporal width (20 fs) of the pulse. As in Figs 1, 2, 3, we are actually dealing with a prethermalization process within the smaller well. The subsequent global thermalization is much slower due to the high barrier between the wells. Considering that 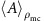 is the only remaining fit parameter in the theory from equations (13) and (20), the agreement with the simulations is remarkably good. In particular, the two very different observables *CT*(*t*) and Δ*P*(*t*) are indeed governed by the same *F*(*t*), as predicted by equations (13) and (20).

In Fig. 5, we compare our theory with the simulations of a different one-dimensional electron model from ref. 59. In doing so, the pertinent temperature *T* has been estimated as follows: the textbook Sommerfeld expansion for *N* electrons in a one-dimensional box yields *E*=*E*_0_[1+(3*π*^2^/8)(*k*_B_*T*/*E*_F_)^2^], where *E* is their total energy, *E*_0_=(1/3)*NE*_F_ the ground-state energy, *E*_F_=(*πℏN*/*gL*)^2^/2*m* the Fermi energy, *L* the box length, *m* the electron mass and *g*:=2*s*+1=2 (*s*=1/2 for electrons). Assuming that the pulse acts solely on the small well implies *N*=16, *L*≃15 nm (ref. 59), and *E*−*E*_0_≃0.045 eV (see Fig. 8a in ref. 59). Altogether, we thus obtain *T*≃170 K.

The remnant ‘fluctuations' of the numerical data in Figs 4 and 5 can be readily explained as finite particle number effects (see Fig. 4 in ref. 32 and Fig. 10 in ref. 59), and their temporal correlations are as predicted below equation (13). The seemingly rather strong fluctuations in Fig. 5 are a fallacy since the systematic changes themselves are very small.

**Figure 6:**
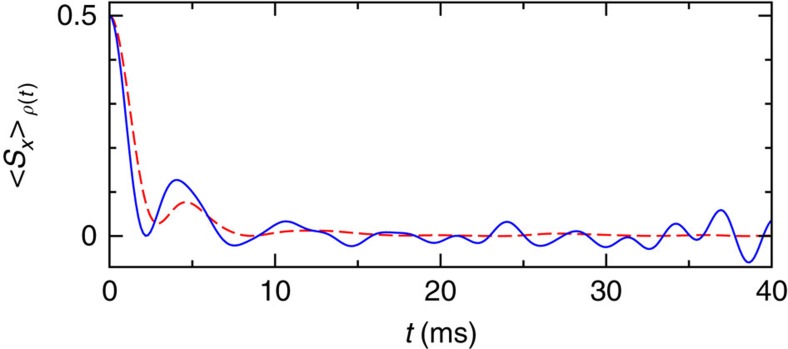
Thermalization of a spin qubit coupled with a bath. Solid: numerical results for the model with seven spin-1/2 degrees of freedom in an external magnetic field from ref. 60: a central spin (qubit) is randomly (and reasonably weakly) coupled with a bath of six spins. The initial state *ρ*(0) is the product of a totally mixed bath state and an eigenstate of the central spin component *S*_*x*_. Depicted are the data from Fig. 2 of ref. 60 for the central spin component *S*_*x*_. Dashed: theoretical prediction (13), (14) and (8). Due to the above-mentioned initial condition and the quite small dimension *D*=2^7^, the approximations (18)–(20), 44, 48 are not very well satisfied by the actual energy eigenvalues *E*_1_, ... , *E*_128_ (kindly provided by the authors of ref. 60). Hence, we have evaluated *F*(*t*) in equation (13) directly via equations (14) and (8).

Next, we turn to the numerical findings for a qubit in contact with a spin bath by the Trauzettel group from ref. 60. The agreement with our theory in Fig. 6 is as good as it possibly can be for such a rather small dimensionality of *D*=2^7^. Indeed, the remaining differences nicely confirm the predictions below equation (13), regarding both their typical order of magnitude 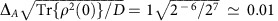 and their temporal correlations (where we exploited that Tr{*ρ*^2^(0)}=2^−6^ for the particular initial condition *ρ*(0) adopted in Fig. 6).

**Figure 7:**
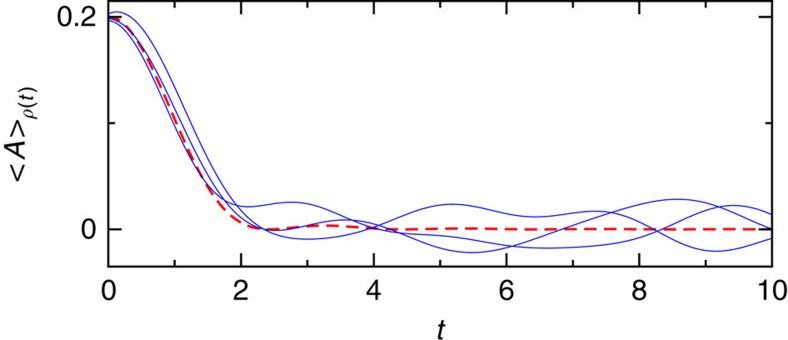
Thermalization in a random matrix model. Solid: numerical results for the random matrix model of the form *H*=*H*_0_+*λV* from ref. 17. Adopting dimensionless units with *ℏ*=1, the *D*=6,000 eigenvalues of *H*_0_ are chosen equidistant with level spacing 8.33 × 10^−5^ (ref. 17). The matrix elements of *A* (observable) and *V* (perturbation) in the basis of *H*_0_ satisfy *A*_*ik*_=(−1)^*k*^*δ*_*ik*_ and 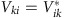. Apart from the latter constraint, the real and imaginary parts of *V*_*ik*_ are independent, normally distributed random numbers. The initial state is 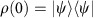, where 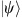 is randomly sampled from the energy shell 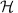 under the constraint 〈*A*〉_*ρ*(0)_≃0.2 (ref. 17). Depicted are three representative numerical realizations for *λ*=7 × 10^−3^ akin to Fig. 1b of ref. 17 (in dimesionless units). Dashed: theoretical prediction (13), (14) and (8). Similarly as in Fig. 6, the numerically obtained energies *E*_1_, ..., *E*_6,000_ were found to satisfy equations (18)–(20) not very well, hence we have directly evaluated equations (8) and (14).

Our final example is Bartsch and Gemmer's random matrix model from ref. 17. Referring to the notation and definitions in the caption of Fig. 7, one readily sees that the considered observable *A* is a conserved quantity for the unperturbed Hamiltonian (*λ*=0). In agreement with our discussion in the section ‘Typical fast relaxation and prethermalization', *A* is therefore still ‘almost conserved' for small *λ* and indeed exhibits a slow, exponential decay towards 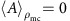 (see Fig. 1a in ref. 17). Upon increasing *λ*, one recovers the much faster, non-exponential decay of our present theory (see Fig. 1b in ref. 17). Unfortunately, the *λ*-value 1.77 × 10^−3^ from Fig. 1b of ref. 17 is still somewhat too small and the eigenvalues *E*_1_, ..., *E*_6,000_ are not any more available (I asked the authors). Therefore, we repeated the numerics from ref. 17 on our own for *λ*=7 × 10^−3^. The resulting agreement with equation (13) in Fig. 7 is very good, and the temporal correlations of the deviations as well as their typical order of magnitude 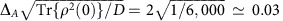 are as predicted below equation (13).

We close with two remarks: first, there is no fit parameter in any of the above examples apart from 〈*A*〉_*ρ*(0)_ in Fig. 4 and 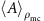 in Figs 4 and 5. Second, especially in the case of the integrable model in Fig. 4, one may question whether the considered system exhibits thermalization in the first place, as is tacitly assumed in equation (13). In Supplementary Note 2, we argue that equation (13) indeed is expected to still remain valid in such cases if 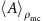 is replaced by the pertinent non-thermal long-time asymptotics (which, in turn, is estimated from the numerical data in Fig. 4).

## Discussion

Our main result (13) implies thermalization in the sense that a generic non-equilibrium system with a macroscopically well-defined energy becomes practically indistinguishable from the corresponding microcanonical ensemble for the overwhelming majority of all sufficiently late times. Apart from the concrete initial and long-time expectation values (that is, 〈*A*〉_*ρ*(0)_ and 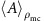 in equation (13)), the temporal relaxation (that is, *F*(*t*) in equation (13)) depends only on the spectrum of the Hamiltonian within the pertinent interval of non-negligibly populated energy eigenstates, but not on any further details of the initial condition or the observable. This represents one of the rare instances of a general quantitative statement about systems far from equilibrium.

The theory agrees very well with a wide variety of experimental and numerical results from the literature (though none of them was originally conceived for the purpose of such a comparison). We are in fact not aware of any other quantitative analytical explanation of those data comparable to ours. Indeed, the usual paradigm to identify and then analytically quantify the main physical mechanisms seems almost hopeless here. In a sense, our present approach thus amounts to a different paradigm: there is no need of any further ‘explanations', since the observed behaviour is expected with overwhelming likelihood from the very beginning, that is, unless there are special *a priori* reasons to the contrary.

Similarly as in refs 46, 49, 50, 51, generic thermalization is found to happen extremely quickly (unless the system's energy or temperature is exceedingly low). Moreover, the temporal decay is typically non-exponential. A main prediction of our theory is that these features should in fact be very common (at least in the form of prethermalization), but often they are unmeasurably fast or they have simply not been looked for so far. Conversely, most of the usually considered observables and initial conditions are actually quite ‘special', namely, exceptionally slow, ‘almost conserved' quantities. A better understanding of those principally untypical but practically very common thermalization processes remains an open problem^49^,^50^,^51^.

## Methods

### Basic matrices

According to the section ‘Analytical results', the unitary *U* represents the basis transformation between the eigenvectors |*n*〉 (*n*=1, ..., *D*) of the Hamiltonian *H* and those of the observable *A*. Denoting the eigenvalues of *A* by *λ*_*ν*_ and the eigenvectors by 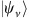 (*ν*=1, ..., *D*), the matrix elements of *U* are thus *U*_*nν*_:=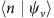. Accordingly, the matrix elements of *ρ*(0) in the basis of *H* are related to those in the basis of *A* via


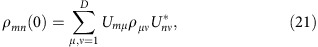


where 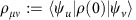. Similarly, the matrix elements of *A* satisfy


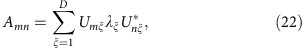


and hence


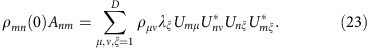


As announced below equation (3), we work (without loss of generality) in a reference frame (or reference basis of 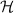) so that only *H* (and thus |*n*〉) depends on *U*, while *A* and *ρ*(0) (and thus 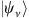) are independent of *U*. Hence, *ρ*_*μν*_ and *λ*_*ξ*_ on the right-hand side of equations (21)–(23), 63, 64 are independent of *U*.

### Derivation of equation (9)

As a simple first exercise, let us average equation (23) over all uniformly (Haar) distributed unitaries *U*, as specified in the section ‘Analytical results'. Since the factors *ρ*_*μν*_*λ*_*ξ*_ on the right-hand side are independent of *U*, we are left with averages over the *U* matrix elements. Such averages have been evaluated repeatedly and often independently of each other in the literature, see, for example, refs 5, 61, 62, 63, a key ingredient being symmetry arguments due to the invariance of the Haar measure under arbitrary unitary transformations. Particularly convenient for our present purposes is the formalism adopted by Brouwer and Beenakker, see ref. 63, and further references therein. The general structure of such averages is provided by equation (2.2) in ref. 63, reading


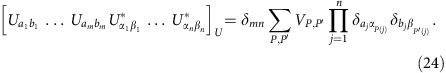


Quoting verbatim from ref. 63, ‘the summation is over all permutations *P* and *P*′ of the numbers 1, ..., *n*. The coefficients *V*_*P*,*P*′_ depend only on the cycle structure of the permutation *P*^−1^*P*′. Recall that each permutation of 1, ..., *n* has a unique factorization in disjoint cyclic permutations (‘cycles') of lengths *c*_1_, ...., *c*_*k*_ (where 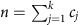). The statement that *V*_*P*,*P*′_ depends only on the cycle structure of *P*^−1^*P*′ means that *V*_*P*,*P*′_ depends only on the lengths *c*_1_, ..., *c*_*k*_ of the cycles in the factorization of *P*^−1^*P*′. One may therefore write 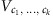 instead of *V*_*P*,*P*′_.' The explicit numerical values of all 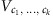 with *n*≤5 are provided by the columns ‘CUE' of Tables II and IV in ref. 63. Further remarks: the labels *m* and *n* in equation (24) have nothing to do with those in equation (23). Equation (24) equals zero unless *m*=*n*. Every label *a*_*j*_ must have a ‘partner', that is, its value must coincide with one of the *α*_*j*_'s and vice versa, since otherwise the product over the Kronecker delta's 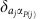 in equation (24) would be zero for all *P*'s. Note that some *a*_*j*_'s may assume the same value, but then an equal number of *α*_*j*_'s also must assume that value. Likewise, every *b*_*j*_ needs a ‘partner' among the *β*_*j*_'s and vice versa.

Adopting the abbreviation


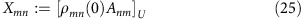


and the renamings *a*_1_:=*m*, *a*_2_:=*n*, *b*_1_:=*μ*, *b*_2_:=*ξ* and *b*_3_:=*ν*, equation (23) yields


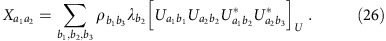


The connection with equation (24) is established via the identifications *α*_1_:=*a*_1_, *α*_2_:=*a*_2_, *β*_1_:=*b*_2_ and *β*_2_:=*b*_3_. Therefore, if *b*_1_≠*b*_2_, then the only potential ‘partner' of *b*_1_ is *β*_2_, and only if their values coincide, that is, *b*_3_=*b*_1_, the corresponding summands may be non-zero. The same conclusion can be drawn if *b*_1_=*b*_2_. We thus can rewrite equation (26) with equation (24) as


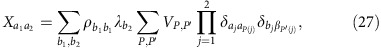


where *β*_1_=*b*_2_ and *β*_2_=*b*_1_.

There are two permutations of the numbers 1 and 2, namely, the identity and one, which exchanges 1 and 2. Denoting them as *P*_1_ and *P*_2_, respectively, and observing that 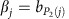, equation (27) can be rewritten as


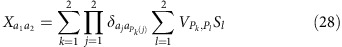



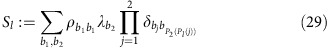


For *l*=1, the two Kronecker delta's in equation (29) both require that *b*_1_=*b*_2_ and hence


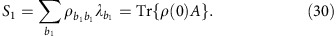


The last equality can be verified by evaluating the trace in the eigenbasis of *A*, see above equation (21). In the same way, one finds that


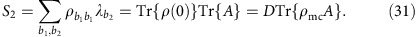


In the last equation, we exploited that Tr{*ρ*(0)}=1 and *ρ*_mc_:=*I*/*D*, see below equation (9). Observing that the two Kronecker delta's in equation (28) equal one if *k*=1 or if *k*=2 and *a*_1_=*a*_2_, the overall result is


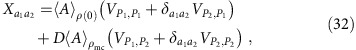


where, as usual, 〈*A*〉_*ρ*(0)_:=Tr{*ρ*(0)*A*} and 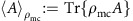.

Finally, the coefficients 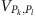 are evaluated as explained below equation (24): if *k*=*l*, then 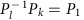 factorizes in two cycles of lengths *c*_1_=*c*_2_=1, that is, 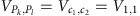. Likewise, if *k*≠*l*, then 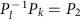 consists of one cycle with *c*_1_=2, that is, 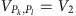. Referring to columns ‘CUE' and rows ‘*n*=2' of Tables II and IV in ref. 63 yields *V*_1,1_=1/(*D*^2^−1) and *V*_2_=−1/[*D*(*D*^2^−1)]. Returning to the original labels *m* and *n* in equation (25), we thus can rewrite equation (32) as


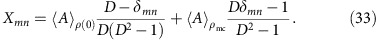


As a consequence, we can infer from equations (4) and (25) that 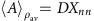 and with equation (33) that


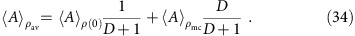


Hence, one readily recovers equation (9).

A relation remarkably similar to our present equation (9), albeit in a quite different physical context, has been previously obtained also in ref. 64 (see equation (2) therein).

### Derivation of equation (11)

Without any doubt, there are much faster ways to obtain equations (33) or (34), 89. The advantage of our present way is that it can be readily adopted without any conceptual differences (albeit the actual calculations become more lengthy) to more demanding cases like


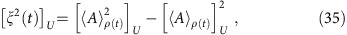


see equation (10).

To evaluate the last term in equation (35), we recast equation (6) with equations (7) and (9) into the form


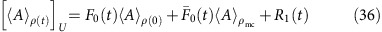



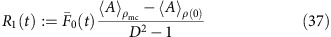



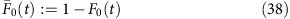



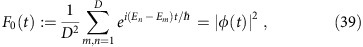


where *φ*(*t*) is defined in equation (8). Similarly as in equation (15), one sees that 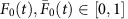 for all *t*. Denoting by *λ*_max_ and *λ*_min_ the largest and smallest among the eigenvalues *λ*_1_, ..., *λ*_*D*_ of *A*, the range of *A* is defined as Δ_*A*_:=*λ*_max_−*λ*_min_. Furthermore, we can and will add a constant to *A* so that *λ*_min_=−*λ*_max_ without any change in the final conclusions below. It readily follows that |*λ*_*ν*_|≤Δ_*A*_/2 for all *ν* and hence that


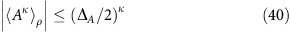


for arbitrary density operators *ρ* and 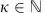. We thus can infer from equation (37) that


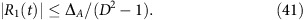


Likewise, one finds upon squaring equation (36) that


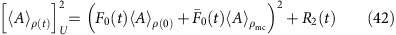



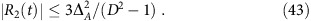


Turning to the first term on the right-hand side of (35), one can infer, similarly as in equations (25) and (26), from (1) and (23) that


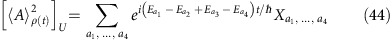



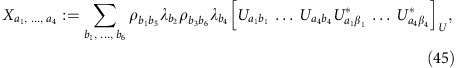


with *β*_1_:=*b*_2_, *β*_2_:=*b*_5_, *β*_3_:=*b*_4_ and *β*_4_:=*b*_6_. Similarly as below equation (26), it follows that only those summands may be non-zero, for which *b*_1_ and *b*_3_ have ‘partners' among *β*_2_ and *β*_4_, and vice versa. This condition can be satisfied in two ways: (i) *b*_5_=*b*_1_ and *b*_6_=*b*_3_; and (ii) *b*_5_=*b*_3_, *b*_6_=*b*_1_ and *b*_1_≠*b*_3_. The latter condition is due to the fact that the case *b*_1_=*b*_3_ is already covered by (i). Exploiting equation (24) and with the abbreviation 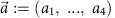 and likewise for 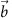, 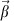 and so on, we thus obtain


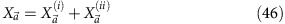



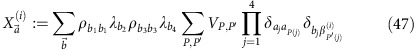



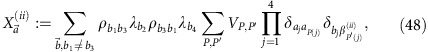


where 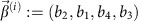 and 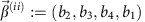.

There are 4!=24 permutations *P* of the numbers 1, 2, 3 and 4. Adopting the shorthand notation [*P*(1)*P*(2)*P*(3)*P*(4)] to explicitly specify a given *P*, these 24 permutations are:


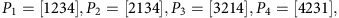



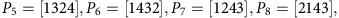



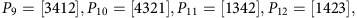



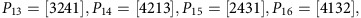



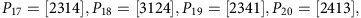



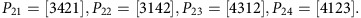


Observing that 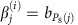 and 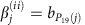 for all *j*=1, ..., 4, it is quite straightforward but very arduous to explicitly carry out the sums over *P*′ and 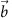 in equations (47) and (48), and the sum over 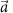 in equation (44), yielding


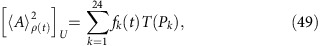


where the functions *f*_*k*_(*t*) are given by


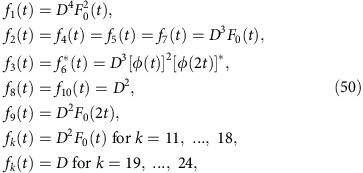


and the coefficients *T*(*P*) are given by


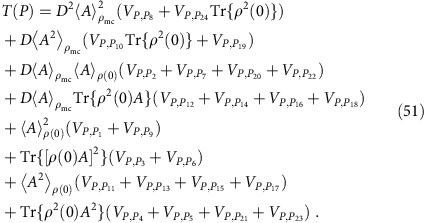


To explicitly evaluate equations (49)–(51), we still need the coefficients 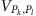 for all *k*,*l*∈{1, ..., 24}. They are obtained as explained below equation (24): defining *j*=*j*(*k*,*l*) implicitly via 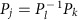, one finds by factorizing each *P*_*j*_ into its disjoint cycles and exploiting Tables II and IV of ref. 63 that 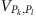 is given by


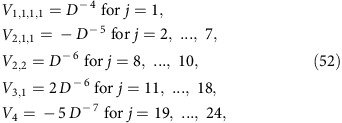


up to correction factors of the form 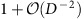 on the right-hand side of each of those relations. One thus is left with finding 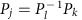 for all 24^2^ pairs (*k*,*l*). To mitigate this daunting task, we have restricted ourselves to those summands in equation (49), which are at least of the order *D*^−1^. Along these lines, one finally recovers with equations (35) the result (11).

### Derivation of equation (13)

While the essential steps in deriving equation (13) have been outlined already in the main text, we still have to provide the details of the statements below equation (13): our first observation is that *R*_1_(*t*) in equation (36) amounts to the systematic (*U* independent) part of the omitted corrections in equation (13), and equation (41) to the bound announced below equation (13).

By means of a straightforward (but again very tedious) generalization of the calculations from the preceding subsection, one finds that


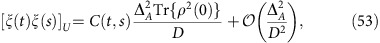


where *C*(*t*, *s*) has the following six properties: first, *C*(*t*, *s*)=*C*(*s*, *t*)=*C*(−*t*,−*s*) for all *t*, *s*. Second, |*C*(*t*, *s*)|≤9 for all *t*,*s*. Third, *C*(*t*, 0)=0 for all *t*. Fourth, *C*(*t*, *s*→0 for |*t*−*s*|→∞, cf. equation (16). Fifth, 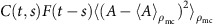 for *t*, *s*→∞. Sixth, given *s*, the behaviour of *C*(*t*, *s*) as a function of *t* is roughly comparable to that of *F*(*t*−*s*) for most *t*.

Though we did not explicitly evaluate the last term in equation (53), closer inspection of its general structure shows that it can be bounded in modulus by 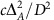 for some *c*, which is independent of *t*, *s*, *D*, *A*, *ρ*(0) and *H*. Moreover, there is no indication of any fundamental structural differences in comparison with the leading and next-to-leading order terms, which we did evaluate. In other words, the last term in equation (53) is expected to satisfy properties analogous to those mentioned below equation (53). Recalling that the purity Tr{*ρ*^2^(0)} satisfies the usual bounds 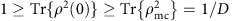, we thus recover the properties of *ξ*(*t*) announced below equation (13).

## Additional information

**How to cite this article:** Reimann, P. Typical fast thermalization processes in closed many-body systems. *Nat. Commun.* 7:10821 doi: 10.1038/ncomms10821 (2016).

## Supplementary Material

Supplementary Information Supplementary Notes 1-2 and Supplementary References.
