# Supplementary material for: Typical fast thermalization processes in closed many-body systems
Source: Nat Commun. 2016 Mar 1;7:10821. doi: 10.1038/ncomms10821 (PMC4773511; doi:10.1038/ncomms10821)
Supplement: Supplementary Information — Supplementary Notes 1-2 and Supplementary References. [file ncomms10821-s1.pdf]

SUPPLEMENTARY INFORMATION

Supplementary Note 1

This note provides a brief account of those previous analytical findings which exhibit some appreciable similarity to ours.

Ref. [1] (see Supplementary References) considers the convergence (for most times) towards some steady state, which is in general different from the microcanonical ensemble. Furthermore, the focus is either on two-outcome measurements, where one of the projectors is of low rank, or the initial state must be an eigenvector of the considered observable. Finally, a role more or less similar to our present randomization via U is played by the assumption that the initial state must be spread over very many energy levels. Within this setting, general upper bounds are obtained for some suitably defined equilibration time scale (as opposed to the (approximate) equality (13) for the entire temporal behavior). Apart from these quite significant differences, the essential conclusions are analogous to ours, namely an extremely rapid relaxation for all above mentioned two-outcome measurements of low rank, as well as for most observables if the initial state is an eigenvector of the observable.

Ref. [2] focuses on subsystem-plus-bath compounds, the total Hilbert space being a collection of many smaller units (e.g. due to a local Hamiltonian on a lattice), and on separable initial states. Under these premises, upper bounds for the subsystem's temporal relaxation are derived, which exhibit some (limited) similarities to our present findings, including the prediction of typically very fast relaxation processes.

Under the additional assumptions that in the latter setup the subsystem is a single qubit, the initial state of the qubit as well as the considered observable are given by Pauli matrices (or the identity), and the environment is in a pure initial state, similar findings as in our present work have been obtained in Ref. [3]. Note that those initial states of the qubit are not very physical and that their linear superposition is not admitted in the findings of [3] due to the non-linearity of the problem.

Refs. [4,5] focus on macroscopic observables with a concomitant projector $P_{\rm neq}$ onto a very small subspace of the "energy shell" $\mathcal{H}$ so that any (normalized) state $|\psi\rangle \in \mathcal{H}$ with $\langle\psi|P_{\rm neq}|\psi\rangle \ll 1$ represents thermal equilibrium. Denoting, similarly as in our present approach, by U the transformation between the bases of the "observable" $P_{\rm neq}$ and the Hamiltonian H, it is then shown that most U result in an extremely quick thermalization for any initial pure state $|\psi(0)\rangle \in \mathcal{H}$. Similarly as in [1] (see above), this conclusion is based on an upper (but arguably rather tight) estimate for the actual temporal relaxation and on similar assumptions about the energy level density $\rho(x)$ as in equations (17)-(20).

In Ref. [6] it is shown that the vast majority of all pure states featuring a common expectation value of some generic observable at a given time will yield very similar expectation values of the same observable at any later time. While in our present approach, $\rho(0)$ and A are kept fixed relatively to each other and U randomizes their constellation relatively to the Hamiltonian H, in Ref. [6] the pair A and H is kept fixed, while $\rho(0)$ is randomly sampled under the additional constraint that it is a pure state with a preset (arbitrary but fixed) expectation value $\langle A\rangle_{\rho(0)}$. Moreover, no quantitative statements about how $\langle A\rangle_{\rho(t)}$ actually evolves in time have been obtained in [6].

Ref. [7] suggests fairly rough relaxation time estimates by exploiting three quite drastic *a priori* assumptions. One of them postulates that the relaxation is monotonous in time, which can in fact not be generally true, see equation (19) and Fig. 6. Apart from that, the obtained estimates are roughly comparable to ours.

Supplementary Note 2

This note compiles some additional remarks and extensions, ordered according to their appearance in the main text.

Regarding section "Setup"

1. In the present paper, we mainly have in mind the examples mentioned below equation (1), i.e., $\mathcal{H}$ represents some microcanonical "energy shell" of a closed many-body system. But similarly as in Refs. [8-10], our main result (13) is actually valid for the more general setup outlined above equation (1), i.e., $\mathcal{H}$ may also represent a more abstract type of "active Hilbert space". For instance, this may be of interest for autonomous systems with few degrees of freedom in the context of semiclassical chaos when the initial state is "spread" over many energy levels.

2. *A priori*, the pertinent Hilbert space of a many-body system is not a microcanonical energy shell $\mathcal{H}$, nor are the Hamiltonian, observables, and system states given by Hermitian operators H, A, and $\rho(t)$ on $\mathcal{H}$ right from the beginning. Rather, the system originally "lives" in a much larger Hilbert space $\mathcal{H}'$ and the Hamiltonian, observables, and system states are given by Hermitian operators H', A', and $\rho'(t)$ on $\mathcal{H}'$. How to go over from the original (primed) to the reduced (unprimed) setup is not very difficult [11-15], but also not entirely obvious:

Similarly as in the main text, we denote by E_n and $|n\rangle$ the eigenvalues and eigenvectors of H', where n runs from 1 to infinity or to some finite upper limit (dimension of $\mathcal{H}'$). Likewise, the corresponding matrix elements of $\rho'(t)$ are denoted as $\rho'_{mn}(t) := \langle m|\rho'(t)|n\rangle$. The key point consist in our assumption below equation (1) that the system exhibit a well defined macroscopic energy, i.e., there exists a microcanonical energy window $I := [E - \Delta E, E]$ so that the level populations $\rho'_{nn}(0)$ are negligibly small for energies E_n outside the interval I. Moreover, we can an will assume that the labels n and

the integer D are chosen so that $E_n \in I \Leftrightarrow n \in \{1,...,D\}$. Next, we denote by $\mathcal{H}$ the subspace spanned by $\{|n\rangle\}_{n=1}^D$, by $P := \sum_{n=1}^D |n\rangle\langle n|$ the projector onto $\mathcal{H}$, and by $H := PH'P$, $A := PA'P$, $\rho(t) := P\rho'(t)P$ the corresponding "restrictions" or "projections" of the original operators. With $|\rho'_{mn}|^2 \leq \rho'_{mm}\rho'_{nn}$ (Cauchy-Schwarz inequality) and the above approximation $\rho'_{nn}(0) = 0$ for $n > D$, it follows that $\rho'_{mn}(0) = 0$ if $m > D$ or $n > D$ and hence that $\rho(0) = \rho'(0)$. Since P commutes with H' and thus with $\mathcal{U}'_t := e^{-iH't/\hbar}$, the original time evolution $\rho'(t) = \mathcal{U}'_t\rho'(0)(\mathcal{U}'_t)^\dagger$ implies that $\rho(t) = \rho'(t)$ for all t, and with $P^2 = P$ it follows that $\rho(t) = \mathcal{U}_t\rho(0)\mathcal{U}_t^\dagger$, where $\mathcal{U}_t := e^{-iHt/\hbar}$. Exploiting the cyclic invariance of the trace and $P^2 = P$ finally yields $\mathrm{Tr}\{\rho'(t)A'\} = \mathrm{Tr}\{\rho(t)A\}$ for all t.

So far, the basic operators H, A, $\rho(t)$ and their descendants $\mathcal{U}_t$ and $\mathrm{Tr}\{\rho(t)A\}$ are strictly speaking still defined on $\mathcal{H}'$ but it is trivial to reinterpret them as being defined on $\mathcal{H}$. In particular, the eigenvalues and eigenvectors of $H : \mathcal{H} \to \mathcal{H}$ are now given by $\{E_n\}_{n=1}^D$ and $\{|n\rangle\}_{n=1}^D$, respectively. While the connection between H and H' and between $\rho(t)$ and $\rho'(t)$ is thus rather trivial, the eigenvalues and eigenvectors of A are in general quite different from those of A'. Nevertheless, all original (primed) expectation values are correctly recovered within the reduced (unprimed) formalism.

Regarding section "Analytical results"

3. A natural intuitive guess is that $\rho_{nn}(0)$ and A_{nn} should be essentially independent of each other in the sense that $[\rho_{nn}(0)A_{nn}]_U$ can be approximated by $[\rho_{nn}(0)]_U\,[A_{nn}]_U$. If so, one could readily conclude from equations (2) and (4) that $\langle A\rangle_{\rho_{\rm av}} = \langle A\rangle_{\rho_{\rm mc}}$, which is nothing else than the leading order approximation of equation (9). In other words, our guess seems right, the essence of equation (9) is intuitively quite obvious, and the last term in equation (9) must be due to weak correlations between $\rho_{nn}(0)$ and A_{nn} for non-equilibrium initial conditions $\langle A\rangle_{\rho(0)}$.

Regarding section "Basic properties of $F(t)$"

4. The essential prerequisite in approximating equation (8) by (17) is that $t/\hbar$ must be much smaller than the inverse mean level distance. Since the energy levels are extremely dense for typical many-body systems, the approximation applies for all experimentally realistic times t. However, the quasi-periodicities of $F(t)$ for extremely large t, inherited from $\phi(t)$ via (7) and (8), usually get lost.

Regarding section "Typicality of thermalization"

5. By similar methods as in the derivation of our main result (13), one can show [11] that for the overwhelming majority of unitaries U the diagonal matrix elements A_{nn} remain very close to their mean value $[A_{nn}]_U = \langle A\rangle_{\rho_{\rm mc}}$, a property also known under the name eigenstate thermalization hypothesis (ETH) [12-14]. It is tempting to argue that a violation of ETH indicates an "untypical" case and hence also (13) will be violated. However, there is no reason why the extremely small subset of U's which violate (13) has any relevant overlap with the extremely small subset of U's which violate ETH. In other words, we expect that equation (13) still applies to the vast majority of ETH-violating systems, i.e., provided their initial condition $\rho(0)$ is still sufficiently "typical" to guarantee thermalization. Numerical examples of such cases are provided, e.g., by Ref. [15].

Vice versa, the findings about typicality of ETH and thermalization from [11,12,16-19] are expected to remain valid even when (13) is violated (thus including cases which do not thermalize as rapidly as predicted by (13)).

An analogous consideration applies to the "level populations" $\rho_{nn}(0)$: They must be negligible outside the microcanonical energy window $[E - \Delta E, E]$, but inside the window they may still be distributed quite "untypically".

6. More abstractly speaking, in order to realize simultaneously an untypical U and a far from equilibrium $\langle A\rangle_{\rho(0)}$, one generally expects that the eigenbases of both A and $\rho(0)$ must be fine-tuned relatively to a given H.

As a consequence, one expects untypically strong correlations between A_{nn} and $\rho_{nn}(0)$ (see also paragraph 3. above). This is confirmed, e.g., by the numerical examples in Refs. [14,20] and is also closely related to the ideas proposed by Peres in Ref. [21].

7. To further scrutinize the untypical U's, we consider subsets S_a consisting of all U's with the extra property that $\langle A\rangle_\omega = a$, where ω is defined below equation (3). One readily sees that for any given a-value, the set S_a still entails the necessary symmetries so that equations (2)-(8) remain valid when re-defining $[\cdots]_U$ as the restricted average over all $U \in S_a$. On the other hand, equation (9) is replaced by $\langle A\rangle_{\rho_{\rm av}} = a$, implied by $\langle A\rangle_\omega = a$ for all $U \in S_a$. Finally, one expects, analogously as in equations (10)-(12), that the fluctuations about the average behavior are typically small for most $U \in S_a$. While a rigorous proof seems very difficult, the intuitive argument is that the subset S_a can be represented as a manifold of fantastically large dimensionality (just one dimension less that for the unrestricted set of all U's due to the extra constraint $\langle A\rangle_\omega = a$). Hence, a similar concentration of measure phenomenon is expected in both cases. Analogously as in (13), the overall conclusion is that

$$\langle A\rangle_{\rho(t)} \;=\; a + F(t)\,\left\{\langle A\rangle_{\rho(0)} - a\right\}$$

should be satisfied in very good approximation for the vast majority of all times t and unitaries $U \in S_a$. Upon

comparison with (13) one sees that if a notably differs from $\langle A\rangle_{\rho_{\rm mc}}$ then most $U \in S_a$ are untypical. On the other hand, any given untypical U is contained in one of the subsets S_a and is thus generically expected to satisfy the above approximation. Remarkably, the time dependence is governed by the same function $F(t)$ for all a.

These considerations justify our comparison of the theory (in the above generalized version) with the integrable model in Fig. 4. In turn, the good agreement with the numerical results in Fig. 4 supports the above arguments.

Supplementary References

[1] Malabarba, A. S. L., Garcia-Pintos, L. P., Linden, N., Farrelly, T. C., & Short, A. J. Quantum systems equilibrate rapidly for most observables. *Phys. Rev. E* **90**, 012121 (2014)

[2] Cramer, M. Thermalization under randomized local Hamiltonians. *New. J. Phys.* **14**, 053051 (2012)

[3] Znidaric, M., Pineda, C., & Garcia-Mata, I. Non-Markovian behavior of small and large complex quantum systems. *Phys. Rev. Lett.* **107**, 080404 (2011)

[4] Goldstein, S., Hara, T., & Tasaki, H. Extremely quick thermalization in a macroscopic quantum system for a typical nonequilibrium subspace. *New. J. Phys.* **17**, 045002 (2015)

[5] Goldstein, S., Hara, T., & Tasaki, H. The approach to equilibrium in a macroscopic quantum system for a typical nonequilibrium subspace. Preprint at http://arxiv.org/abs/1402.3380 (2014)

[6] Bartsch, C. & Gemmer, J. Dynamical typicality of quantum expectation values. *Phys. Rev. Lett.* **102**, 110403 (2009)

[7] Monnai, T. Generic evaluation of relaxation time for quantum many body systems: analysis of system size dependence. *J. Phys. Soc. Jpn.* **82**, 044006 (2013)

[8] Popescu, S., Short, A. J., & Winter, A. Entanglement and the foundations of statistical mechanics. *Nature Phys.* **2**, 754-758 (2006)

[9] Popescu, S., Short, A. J., & Winter, A. The foundations of statistical mechanics from entanglement: Individual states vs. averages. Preprint at http://arxiv.org/abs/quant-ph/0511225 (2005)

[10] Linden, N., Popescu, S., Short, A. J., & Winter, A. Quantum mechanical evolution towards equilibrium. *Phys. Rev. E* **79**, 061103 (2009)

[11] von Neumann, J. Beweis des Ergodensatzes und des H-Theorems in der neuen Mechanik. *Z. Phys.* **57**, 30-70 (1929) [English translation by Tumulka, R. Proof of the ergodic theorem and the H-theorem in quantum mechanics. *Eur. Phys. J. H* **35**, 201-237 (2010)]

[12] Goldstein, S., Lebowitz, J. L., Tumulka, R., & Zhangì, N. Long-time behavior of macroscopic quantum systems: commentary accompanying the english translation of John von Neumann's 1929 article on the quantum ergodic theorem. *Eur. Phys. J. H* **35**, 173-200 (2010)

[13] Goldstein, S., Lebowitz, J. L., Mastrodonato, C., Tumulka, R., & Zhangì, N. Approach to thermal equilibrium of macroscopic quantum systems. *Phys. Rev. E* **81**, 011109 (2010)

[14] Goldstein, S., Lebowitz, J. L., Mastrodonato, C., Tumulka, R., & Zhangì, N. Normal typicality and von Neumann's quantum ergodic theorem. *Proc. R. Soc. A* **466**, 3203-3224 (2010)

[15] Reimann, P. Generalization of von Neumann's approach to thermalization. *Phys. Rev. Lett.* **115**, 010403 (2015)

[16] Deutsch, J. M. Quantum statistical mechanics in a closed system. *Phys. Rev. A* **43**, 2046-2049 (1991)

[17] Srednicki, M. Chaos and quantum thermalization. *Phys. Rev. E* **50**, 888-901 (1994)

[18] Rigol, M., Dunjko, V., & Olshanii, M. Thermalization and its mechanism for generic isolated quantum systems. *Nature* **452**, 854-858 (2008)

[19] Rigol, M. & Srednicki, M. Alternatives to eigenstate thermalization. *Phys. Rev. Lett.* **12**, 110601 (2012)

[20] Gogolin, C., Müller, M., & Eisert, J. Absence of thermalization in nonintegrable systems. *Phys. Rev. Lett.* **106**, 040401 (2011)

[21] Peres, A. Ergodicity and mixing in quantum theory I. *Phys. Rev. A* **30**, 504-509 (1984)
